# Assessing the industry 4.0 strategies for a steel supply chain: SWOT, game theory, and gap analysis

**DOI:** 10.1016/j.heliyon.2024.e41374

**Published:** 2024-12-18

**Authors:** Sima Motallebi, Mostafa Zandieh, Akbar Alem Tabriz, Erfan Babaee Tirkolaee

**Affiliations:** a Department of Industrial Management and Information Technology, Faculty of Management and Accounting, Shahid Beheshti University, Tehran, Iran; b Department of Industrial Engineering, Istinye University, Istanbul, Turkey; c Department of Industrial Engineering and Management, Yuan Ze University, Taoyuan, Taiwan; d Department of Mechanics and Mathematics, Western Caspian University, Baku, Azerbaijan

**Keywords:** Industry 4.0, Supply chain, Steel industry, Game theory, Gap analysis

## Abstract

The recent adoption of modern technologies has led to the fourth industrial revolution or Industry 4.0 (I4.0). The supply chain of large industries, such as steel, is of significant importance in the economies of countries. According to data published by the National Iranian Steel Company, the steel industry is one of the most vital sectors in Iran, contributing about 1 % to its Gross Domestic Product (GDP). Therefore, implementing I4.0 in the steel supply chain is a highly significant and practical endeavor, with the potential to drive considerable advancements in this industry. This study develops an efficient integrated framework to identify and evaluate the most critical factors influencing the implementation of I4.0 in the supply chains of large industries. The study also reviews the most effective policies for implementing these factors and analyzes the gap between the current state of the steel supply chain in Iran and the desired state within the framework of I4.0. The components of I4.0 are determined based on a review of existing literature and interviews with experts from the Iranian steel industry, utilizing the fuzzy Delphi method. Afterward, Strengths, Weaknesses, Opportunities, and Threats (SWOT) policies are developed. The optimal combination of policies for implementing I4.0 in the Iranian steel supply chain is identified using game theory. Finally, a gap analysis is conducted between the current state of the industry and the desired state. The critical gap analysis revealed that the current state of the supply chain significantly lags behind the desired I4.0 goals, particularly in terms of infrastructure and supply chain balancing. The findings highlight a stark contrast between the current performance score of 0.16 and the target score of 0.84, underscoring the substantial improvements needed to realize I4.0 within this sector.

## 1 Introduction

**Fig. 1:**
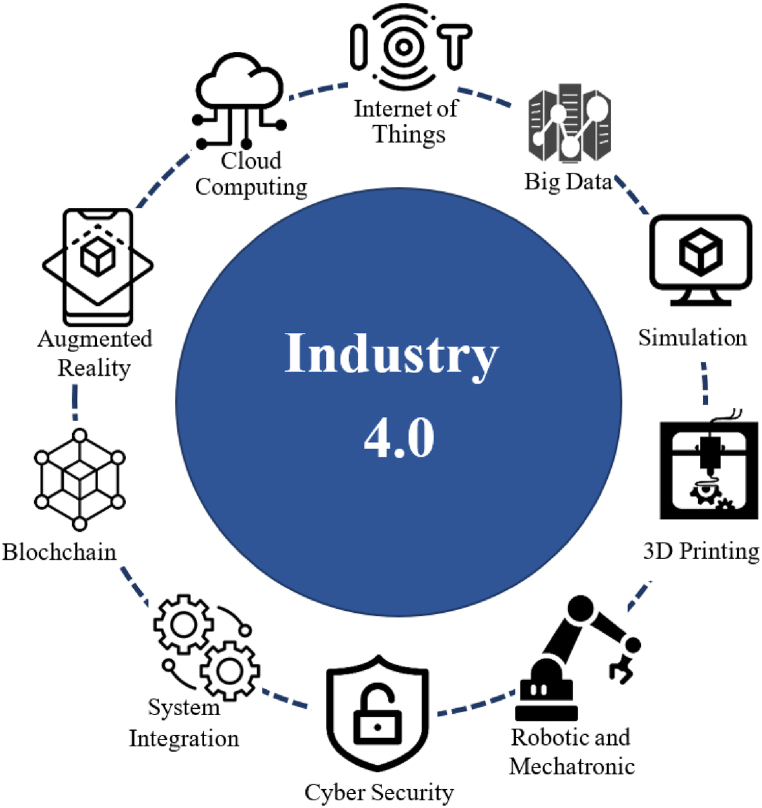
I4.0 technologies.

The effective functioning of supply chains in large industries is crucial to the economic well-being of nations. The complex relationships between producers and consumers, along with varying levels of involvement, make managing and controlling these supply chains highly challenging [1]. Recent advancements in information technology have driven manufacturing companies to leverage expertise and capabilities to establish efficient and consistent supply chains that meet customer needs. Industry 4.0 (I4.0), a convergence of technologies such as cloud computing, the Internet of Things (IoT), blockchain, and other innovations (see Fig. 1), has emerged as a transformative force in this context. Originating in Germany in 2012, I4.0 marks the fourth industrial revolution, shifting economic policies towards the development of innovative technologies. This paradigm prioritizes optimizing capabilities, efficiency, and overall performance over traditional expectations of organizational excellence [2,3]. I4.0 envisions interconnected networks of intelligent factories that exchange information and autonomously control various processes. In this cyber-physical system, products are treated as distinct, active entities moving along a defined path within the supply chain.

The supply chain of large industries, such as steel, plays a pivotal role in national economies. In Iran, the steel industry is a significant pillar of the economy, contributing approximately 1 % of the country's Gross Domestic Product (GDP), according to data from the National Iranian Steel Company. The Iranian government's 2025 vision for steel production aims to increase output from 35 million tons to 55 million tons. Furthermore, Iran's broader economic policies emphasize reducing dependence on oil through industrial progress, aligning with the ambitious goals set in the 2025 vision. Implementing I4.0 in the Iranian steel supply chain is essential for achieving these objectives.

Recent studies have explored the impact of I4.0 on supply chain management. For instance, a 2024 study by Lu et al. examined circular supply chains enabled by the integration of circular economy and I4.0 paradigms. The study highlights key trends and provides a theoretical framework to assist practitioners in transitioning to circular supply chains [4]. Another study by Patil et al. (2023) investigated the readiness factors for sustainable supply chain management in the context of big data and I4.0, identifying 17 potential readiness factors and prioritizing them using a fuzzy best-worst method [5]. Additionally, Gharaibeh et al. (2024) evaluated the status of I4.0 in supply chains by thematically analyzing the literature and mapping research trends [6].

Despite the growing body of research on I4.0, there remains a significant gap in understanding the specific strategies and challenges associated with its implementation in the Iranian steel supply chain. This study aims to address this gap by.

• Identifying the most critical factors influencing the implementation of I4.0 in the supply chain of large industries.
• Developing and evaluating the most significant policies for implementing these factors.
• Analyzing the gap between the current state of the Iranian steel supply chain and the desired state of I4.0.

By addressing the mentioned research gap, this study contributes to a deeper understanding of the potential benefits and challenges of I4.0 adoption in the steel supply chain, offering valuable insights for policymakers and industry practitioners. The objective is to identify and evaluate the most influential factors in the deployment of I4.0 within the supply chain of large industries. We then examine the key policies for implementing these factors, followed by an analysis of the gap between the current state of the Iranian steel supply chain and the desired state of I4.0.

This study focuses on the Iranian steel supply chain, employing a mixed-method approach that integrates SWOT analysis, game theory, and gap analysis. By addressing key factors and strategic policies, the research aims to bridge the gap between the current state of the supply chain and Industry 4.0 objectives.

The remaining sections of this manuscript are organized as follows. Section 2 presents a literature review and analysis of relevant articles, which serves as the basis for identifying the components of I4.0. Section 3 details the methodology employed in this study. The results and discussion are provided in Section 4, where we use the fuzzy Delphi method to identify the SWOT components of I4.0 and develop combined policies based on expert insights from the Iranian steel industry. We then apply game theory to determine the optimal combination of policies for implementation in the Iranian steel supply chain, followed by a gap analysis between the desired and current states. Section 5 concludes the study and outlines directions for future research.

## 2 Literature survey

Based on the goals and research gaps, in this section, we first conduct a bibliometric analysis of our subject to achieve a general consensus on the topic. Following this, we categorize recent research based on the results of the bibliometric analysis, allowing us to identify and classify the components of I4.0 according to this literature review.

### 2.1 Bibliometric analysis

The concept of I4.0 was first introduced in Germany in 2012 and has since become a focal point of numerous research studies. These studies have explored its multifaceted impacts and implications across various aspects of the industrial landscape.

**Fig. 2:**
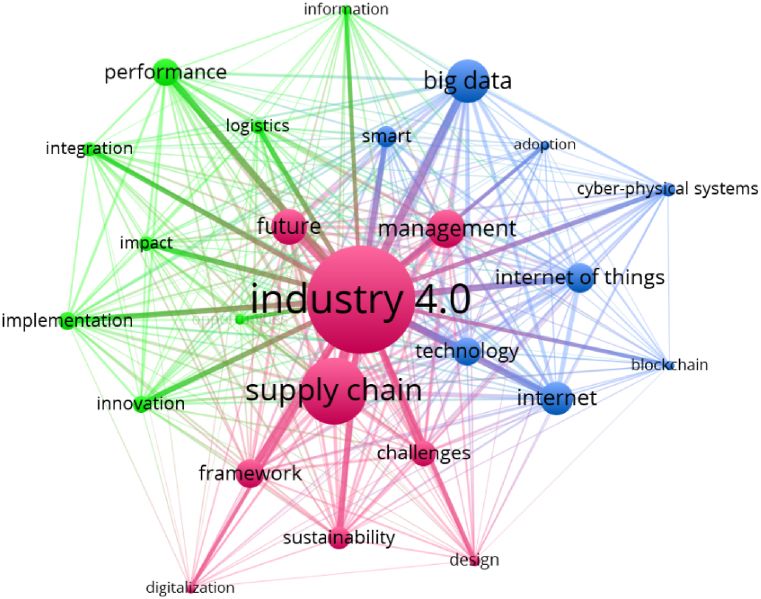
Keywords co-occurrences network.

At first, we conducted a bibliometric analysis using metadata from the Web of Science database. A total of 416 documents were selected for examination. These documents, featuring 1311 keywords, were based on 26,121 references from 150 published journals. To identify the most significant concepts in the field of I4.0 within the supply chain, we generated a keyword co-occurrence network using VOSviewer software (see Fig. 2).

In this network, keywords related to the research field are connected based on their co-occurrences, representing the conceptual space of the subject. Both Author Keywords and Keywords Plus (keywords created by Web of Science) were used to construct this network. The visual representation illustrates the conceptual structure of I4.0 in the supply chain, with each keyword requiring a minimum of 20 occurrences to be included in the image.

Based on the analysis, out of 1760 keywords, 34 have been incorporated into the network. This network consists of three clusters.

• The first cluster, shown in red, encompasses the core concepts of “Industry 4.0” and “supply chain” which are the primary topics in this field, forming the largest nodes in the network.
• The second cluster, represented in blue, pertains to I4.0 technologies. The most prominent nodes in this cluster include “Cyber-Physical Systems”, “Big Data”, “Internet of Things”, “Technology” and “Internet”.
• The third cluster, depicted in green, includes nodes such as “Performance”, “Innovation” and “Implementation”, reflecting the managerial dimension and strategies for adopting I4.0 in the supply chain.

**Fig. 3:**
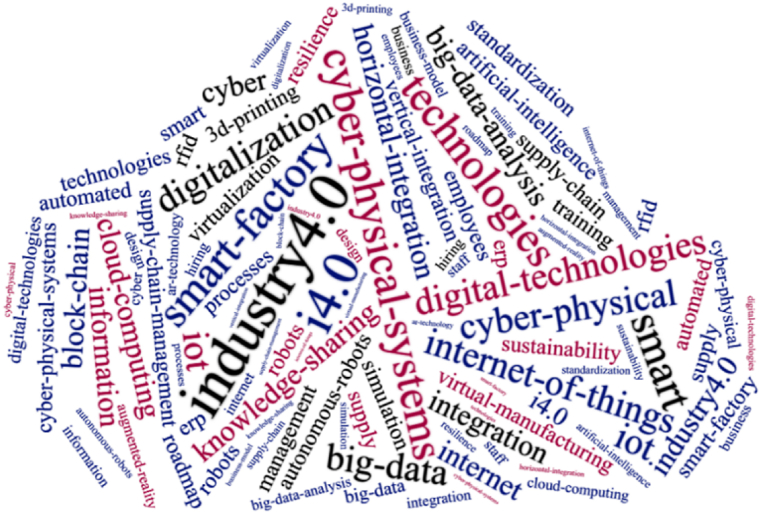
Keywords cloud map.

Additionally, a keyword cloud is provided in Fig. 3, which has been generated using 50 keywords.

**Fig. 4:**
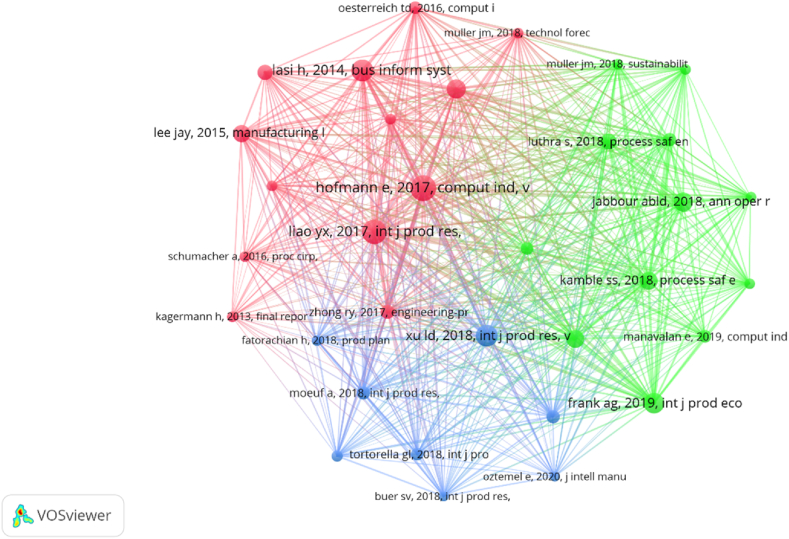
Co-citation network.

Afterward, we conducted a co-citation analysis to establish a general classification of previous research. In this analysis, two documents are considered connected in the co-citation network when they are referenced together in other studies. Fig. 4 illustrates the co-citation network, which consists of 45 documents, each cited at least 40 times. Accordingly.

• First cluster, represented in red, emphasizes the cyber aspects of I4.0, including areas like cloud computing, simulation, and ERP, as well as how these tools can be utilized to control the supply chain.
• Second cluster, marked in green, focuses on assessing the physical performance of I4.0 technologies and the challenges associated with using them to enhance industrial efficiency.
• Third cluster, highlighted in blue, comprises articles primarily centered on the future of I4.0, its management, and strategic direction. These articles explore upcoming trends, the current state of I4.0, its strategic roadmap, and the processes of adoption and implementation.

### 2.2 General classification of previous studies

By reviewing the identified clusters in last part, and analyzing the key articles within each, a general categorization of previous literature was established.

**Fig. 5:**
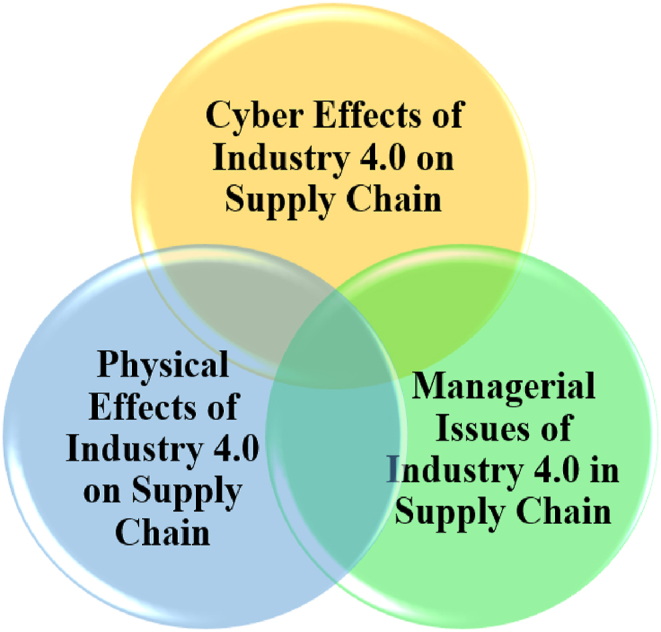
General classification of previous articles.

The recent research works can generally be categorized into three broad classes (see Fig. 5).

#### 2.2.1 Cyber effects of I4.0 on supply chain

These studies primarily focus on the digital and technological aspects of I4.0, such as data analytics and advanced tracking systems. Tjahjono et al. (2017) explored the impact of I4.0 on supply chains, defining the product as an active, unique, and identifiable entity that follows its path within the supply chain. In I4.0, the roles of suppliers, producers, and customers are fully transparent and traceable from order placement to the end of the product lifecycle [7]. This study emphasizes how I4.0 technologies, such as data analytics, enhance supply chain visibility and transparency. While primarily focused on cyber aspects, it also touches on the physical effects of improved visibility and transparency. Rudolph and Emelmann (2017) compared the order process, pricing, and various production costs associated with new I4.0 technologies, highlighting the role of digital technologies in streamlining these processes [8]. Ivanov (2019) examined the relationship between I4.0, big data, and advanced tracking systems, stressing the importance of data-driven insights in managing supply chain risks [9]. The study by D'Oliveira-Dias et al. (2023) investigated the interaction between I4.0 base technologies and agile/lean supply chain strategies, focusing on their support for operational performance [10]. Younis and Wuni (2023) conducted a scientometric analysis and critical review of the application and role of I4.0 enablers in supply chain management, emphasizing how digital technologies enhance efficiency and decision-making [11].

#### 2.2.2 Physical effects of I4.0 on supply chain

These studies focus on the tangible, physical impacts of I4.0 on the supply chain, including production systems, infrastructure, and logistics. Brant and Sundaram (2015) examined the performance of a production system based on large metal structures, analyzing factors such as the accuracy of implementing instructions, reliability, data sufficiency, and production time and efficiency [12]. Luthra and Kumar Mangla (2018) identified challenges related to the physical implementation of I4.0 in the supply chain, including infrastructure, technology adoption, and workforce skills [13]. Fatorachian and Kazemi (2021) explored the impact of I4.0 on supply chain performance, presenting their findings as a practical conceptual framework. They concluded that I4.0 technologies can optimize supply chain performance. This study could be categorized under both Physical Effects and Managerial Issues, as it addresses the physical impact of I4.0 on supply chain performance and the managerial implications for process optimization [14]. Caiado (2021) proposed a model for achieving I4.0 maturity in the supply chain, focusing on physical aspects [1]. Jaouhari et al. (2023) examined the contribution of I4.0 technologies to achieving net-zero supply chain performance through a review and introspective analysis, offering a roadmap based on identified knowledge gaps between I4.0 and sustainability [15].

#### 2.2.3 Managerial issues of I4.0 in supply chain

These studies focus on the challenges and opportunities related to managing the implementation of I4.0 in the supply chain, including decision-making, sustainability, and organizational readiness. Taddei et al. (2022) conducted a systematic literature review on circular supply chains enabled by the convergence of circular economy and I4.0 paradigms. This study highlights the management challenges and opportunities involved in transitioning to circular supply chains within the context of I4.0 [16]. Patil et al. (2023) identified key readiness factors for sustainable supply chain management in I4.0, stressing the importance of management practices like data analysis and risk assessment [5]. Zayat et al. (2023) reviewed the application of Multiple Attribute Decision-Making (MADM) methods across various I4.0 components, emphasizing the significance of effective management tools and techniques [17]. Torkayesh et al. (2022) proposed a hybrid MADM approach for adopting I4.0-based technologies in Spain's transport and mobility sector, underscoring the necessity of sound management decisions in the implementation process [18].

The reviewed studies on I4.0 in supply chains provide valuable insights but also reveal certain limitations. For instance, while some studies focus on enhancing transparency, cost efficiency, and risk management through digital technologies, they do not thoroughly explore the physical challenges of implementation. Others emphasize the performance of production systems but offer limited insights into the technological hurdles, managerial implications, and infrastructural challenges of adopting I4.0.

This study aims to bridge these gaps by offering an integrated framework that addresses both cyber and physical impacts while also considering managerial issues, thereby providing a more holistic view of I4.0 implementation in supply chains.

While prior studies, such as those by Patil et al. (2023) and Gharaibeh et al. (2024), focused on readiness factors and thematic mappings of Industry 4.0, this work uniquely integrates game theory with gap analysis to propose actionable strategies tailored to the Iranian steel supply chain. Furthermore, by evaluating the disparities between the current and desired states, this study contributes insights into the infrastructural and strategic prerequisites for successful Industry 4.0 implementation.

### 2.3 I4.0 components

**Fig. 6:**
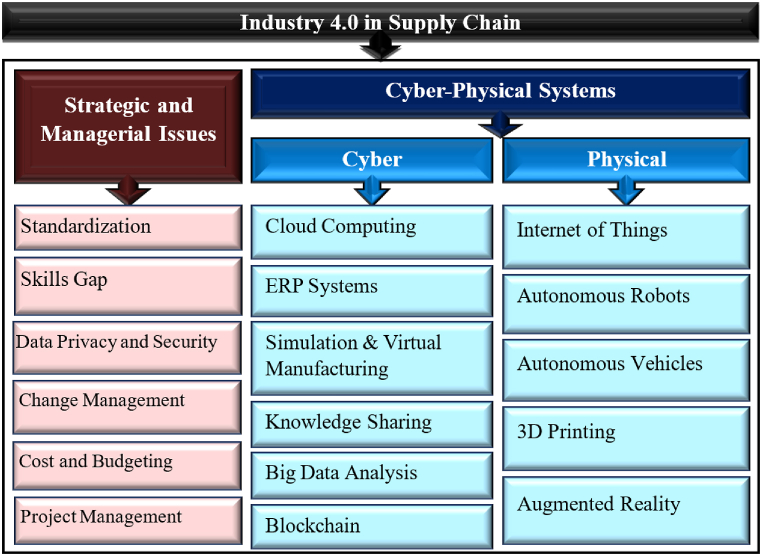
I4.0 components.

Based on the literature review and analysis of the extracted articles, the components of I4.0 are illustrated in Fig. 6 Ultimately, we categorized the components of I4.0 into two main groups according to the studies: (i) Cyber-Physical Systems and (ii) Strategic and Managerial Issues.

#### 2.3.1 Strategic and managerial issues

There are strategic and managerial issues that must be considered for the successful implementation of I4.0. While I4.0 holds tremendous potential for improving factories, there are also strategic and managerial issues that must be considered for successful implementation. These include the lack of standardization, the skills gap, data privacy and security, organizational change management, cost and budgeting, and effective project management. By addressing these issues, factories can ensure a safe transition to I4.0 and reap the benefits of advanced technologies in their operations (see Table 1).

**Table 1:** Strategic and managerial issues in I4.0.

Strategic and Managerial Issues	Strategic Issues	Lack of Standardization	The lack of standardization is a significant strategic issue, as it makes it difficult for factories to integrate these technologies into their existing processes [19].
		Skill Gap	The skill gap between existing workers and new technologies necessitates significant investment in training and reskilling programs [20].
		Data Privacy and Security	I4.0 technologies raise data privacy and security concerns, requiring factories to implement robust data management systems to protect sensitive information [21].
	**Managerial Issues**	Organizational Change Management	Implementing I4.0 demands effective communication, stakeholder engagement, and leadership to ensure a smooth and safe transition [22].
		Cost and Budgeting	Factories must allocate adequate budgets for the deployment and maintenance of I4.0 technologies, guided by effective cost-benefit analysis and budgeting processes [22].
		Project Management	Clear project objectives, timelines, budgets, and responsibilities, along with regular monitoring and evaluation of progress, are critical for the successful deployment of I4.0 technologies in factories [23].

#### 2.3.2 Cyber-physical systems

Cyber-physical systems integrate computational and physical capabilities, enabling interaction between physical objects, such as humans and machines. I4.0 aims to connect these physical objects, allowing them to share data and information through the use of sensors and network connections. The foundation of I4.0 lies in continuous connectivity via the Internet, creating a platform that facilitates ongoing interaction and information exchange between humans and machines, as well as among machines themselves [24].

Cyber-physical systems are divided into two main components: physical systems and cyber systems, which interact with one another. The combination of these systems allows for the seamless integration of computational and physical processes, resulting in intelligent and efficient systems [25].

Table 2 provides a detailed explanation of the components of cyber-physical systems.

**Table 2:** Cyber-physical systems in I4.0.

**Cyber-Physical Systems**	**Cyber Systems**	Cloud Computing	Cloud computing is an IT services delivery model where resources are made available over the Internet, allowing users to access the services they need without owning the underlying infrastructure or equipment [26].
		ERP Systems	ERP systems help organizations integrate and manage I4.0 technologies by providing a centralized platform for data management, automation, and decision-making [27].
		Simulation and Virtual Manufacturing	Simulation and virtual manufacturing involve the use of computer-based models and simulations to design, test, and optimize products, processes, and production systems [28].
		Knowledge Sharing	I4.0's knowledge sharing focuses on the real-time exchange of information, utilizing advanced technologies to drive innovation, efficiency, and competitiveness in the industry [29].
		Big Data Analysis	Big data analysis offers insights from large datasets to support data-driven decision-making regarding customer behavior, market trends, and operational efficiency, ultimately leading to increased profitability [30].
		Blockchain	Blockchain technology enhances supply chain transparency, accountability, and efficiency by providing a decentralized ledger for tracking transactions and product movements. It reduces costs, streamlines processes, minimizes fraud, and automates contract execution, leading to increased trust and competitiveness [31].
	**Physical Systems**	Internet of Things	IoT is a network of physical objects that connect to the Internet and communicate with each other, revolutionizing how people live and work [32].
		Autonomous Robots	Autonomous robots operate independently, detect changes in their environment, make decisions, and execute tasks. They are efficient, work without rest, and improve safety in hazardous workplaces [33].
		Autonomous Vehicles	Autonomous vehicles utilize sensors, cameras, GPS, and algorithms to navigate without human intervention [34].
		3D Printing	3D printing involves creating three-dimensional objects by layering material based on computer-aided design (CAD) software. It is widely used in manufacturing, construction, and healthcare due to its cost-effectiveness and efficiency [35].
		Augmented Reality	Augmented reality integrates virtual objects and information into the real world using technology, enhancing human perception by overlaying digital information and images onto the user's view of the physical environment [36].

Finally, by considering both Cyber-Physical Systems and Strategic and Managerial Issues, we can leverage the insights gained from this section to address the research questions in the subsequent sections.

After reviewing the literature, we cannot find a comprehensive study that outlines all the key factors influencing the impact of I4.0 on the supply chain. Therefore, one of the contributions of our research is the examination of these factors, which is presented in this section.

Following this, in the subsequent sections, our next contribution is a comprehensive SWOT analysis, through which we complete these identified factors and develop strategies for implementing these technologies in large supply chains. This research can be useful for future academic researchers and industry practitioners to implement I4.0.

## 3 Methodology

**Fig. 7:**
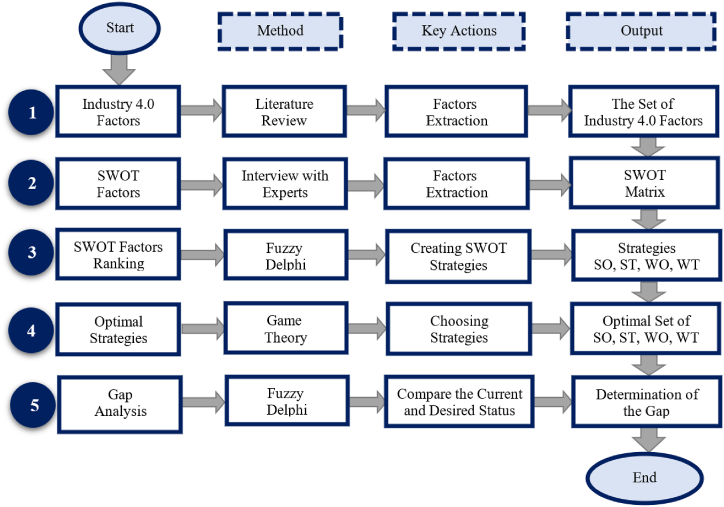
Research methodology.

In this section, we provide a comprehensive overview of the research methodology, which is designed to rigorously examine and analyze the components of I4.0 within the context of the Iranian steel supply chain. Fig. 7 presents the research methodology framework. Subsequently, Each section will be explained in detail.

### 3.1 Identification of I4.0 factors

In the previous section, the components of I4.0 were categorized into Cyber-Physical Systems and Strategic and Managerial Issues through a comprehensive literature review.

### 3.2 Identification of SWOT factors

Building on the insights from the literature review and informed by interviews with domain experts, we identified key factors relevant to the SWOT analysis. Specifically, 15 items were extracted for each category: strengths, weaknesses, opportunities, and threats, which are explained in detail in Section 4.

### 3.3 SWOT factors ranking

To gain a deeper understanding of these factors, the fuzzy Delphi method was employed, a robust technique recognized for effectively aggregating expert opinions. A carefully selected panel of experts, chosen for their expertise in the field, was consulted. These experts evaluated and rated the identified factors using a 5-point Likert scale, providing valuable insights into the significance of each factor. The aggregated expert opinions were then distilled into a concise set of six items for each aspect of the SWOT analysis.

### 3.4 Determination of optimal strategies

Subsequently, based on the refined SWOT factors, we formulated specific policies designed to address the identified issues effectively. These policies outline the strategies and actions required to mitigate weaknesses and threats while leveraging strengths and opportunities. This step involved consultation with research experts to ensure the policies’ comprehensiveness and viability. To determine the optimal combination of these policies for implementation within the Iranian steel supply chain, we utilized game theory. Game theory offers a powerful framework for strategically analyzing decision-making in complex environments [37]. By applying game theory, we aimed to identify the policies that would best serve the interests of the Iranian steel supply chain, considering various scenarios and stakeholders. This approach was chosen because it enables the evaluation of cooperative behavior among the policies and helps identify the most effective combinations for implementing I4.0 in the Iranian steel supply chain.

### 3.5 Gap analysis

Finally, a gap analysis was conducted, comparing the desired state, as determined by the research, with the current state of the Iranian steel supply chain. In this phase, the fuzzy Delphi method was applied to achieve a more nuanced assessment that accounts for varying degrees of desirability and feasibility.

By following this comprehensive methodology, the study aims to provide a solid foundation for determining SWOT strategies. This approach ensures that the integration of I4.0 into the supply chain of large industries, particularly the Iranian steel sector, is both effective and well-informed.

## 4 Results and discussion

### 4.1 SWOT factors

SWOT analysis is a strategic management tool used to identify an organization's strengths, weaknesses, opportunities, and threats. It offers a comprehensive overview of both internal and external factors influencing an organization's performance and aiding in developing effective strategies. In recent years, SWOT analysis has become increasingly popular due to its simplicity, flexibility, and ease of application across various contexts. Numerous studies have emphasized the significance of SWOT analysis in strategic management.

Given that Iran encompasses the entire production cycle of the steel industry, (from iron ore extraction and steel production to partial consumer goods production and recycling) its future holds paramount significance for the country's macroeconomic landscape. This highlights the steel industry as the central focus of our research. The steel industry plays a crucial role in driving the development and enhancement of related sectors. For Iran, articulating a forward-looking vision for steel production is imperative for policymakers and decision-makers.

Considering the core characteristics of I4.0, many of the sector's challenges can be addressed through technological adoption. Therefore, our research aims to explore the gap between I4.0 and the Iranian steel supply chain. The research involves insights from 12 industry managers actively engaged in various industrial complexes, including Isfahan Mobarakeh Steel (Isfahan), Iran Alloy Steel (Yazd), Chadormelo Industrial Complex (Ardakan), Arfa Iron and Steel (Ardakan), Golgohar Industrial Complex (Sirjan), Jahan Steel (Sirjan), Iranian Zarand Steel Factory (Kerman), and Sani Kaveh Steel Factory (Tehran).

Based on the factors extracted from the literature and expert interviews, we have identified the SWOT factors relevant to the implementation of I4.0 in the steel supply chain as follows.

#### 4.1.1 Strengths (S)

The implementation of I4.0 in the steel supply chain offers numerous strengths. We conducted a SWOT analysis with research experts to identify existing strengths in the supply chain processes and to determine how the adoption of I4.0 technologies could further enhance these strengths. Table 3 lists 15 strengths associated with I4.0 that can be leveraged in the Iranian steel supply chain.

**Table 3:** Strengths presented by I4.0.

Strengths		
1	Compatibility	I4.0 enhances the ability of companies to share and utilize each other's machinery and equipment with similar functions more effectively.
2	Decentralization	I4.0 empowers machines, personnel, and small businesses to make faster, data-driven decisions.
3	Real-time Responsiveness	I4.0 accelerates response times, allowing machines to adapt to customer needs more quickly and efficiently.
4	Modularity	I4.0 facilitates easy adjustments in the production process to accommodate changes in product design or seasonal demand fluctuations.
5	Service Orientation	Internet of Things (IoT) enables seamless interaction between companies, individuals, and Cyber-Physical Systems (CPSs), enhancing the value delivered across the supply chain.
6	Efficiency Improvement	Technologies such as automation, robotics, and IoT within I4.0 boost supply chain efficiency, reducing energy consumption and minimizing the use of raw materials.
7	Productivity Improvement	Integration of smart technologies in I4.0 enhances productivity and lowers costs by optimizing and streamlining processes.
8	Flexibility	I4.0 technologies provide greater flexibility in production and supply chain operations, allowing organizations to swiftly adapt to changes in demand.
9	Customer Satisfaction	By integrating customers into the production process through I4.0 networks and ensuring personalized, timely delivery of goods, customer satisfaction is significantly enhanced.
10	Data Management	Incorporation of Big Data and advanced analytics into the supply chain improves decision-making and enhances overall data management.
11	Real-Time Monitoring	Use of sensors and connected devices enables continuous real-time monitoring and tracking of goods throughout the supply chain.
12	Visibility and Traceability	I4.0 leverages IoT, Big Data, and AI to improve supply chain visibility and traceability by monitoring and analyzing data in real time, allowing for swift identification and resolution of potential issues.
13	Decision-Making	I4.0 enhances decision-making through data-driven insights, leading to more informed and effective outcomes.
14	Sustainability Improvement	I4.0 promotes sustainability by optimizing resources and reducing waste, contributing to a more eco-friendly supply chain.
15	Supply Chain Security	Technologies such as blockchain, IoT, and advanced data analytics within I4.0 enhance supply chain security by ensuring secure and transparent tracking, reducing the risks of fraud, counterfeiting, and theft.

#### 4.1.2 Weaknesses (W)

The implementation of I4.0 in the supply chain can also introduce certain weaknesses. To identify these, we conducted a SWOT analysis to pinpoint existing weaknesses in the supply chain processes and evaluate how I4.0 technologies might improve these issues. Table 4 lists 15 potential weaknesses that I4.0 could present for the supply chain of the Iranian steel industry.

**Table 4:** Weaknesses presented by I4.0.

Weaknesses		
1	Operator Training	Training operators and enhancing their digital competencies can be both costly and time-intensive.
2	Upskilling	Equipping operators with new skills and transforming the workforce is essential for effective management of digital tasks.
3	Data Sharing	Collaboration and data exchange among competing industries are crucial for success.
4	Implementation Costs	Implementing I4.0 technologies can be expensive and requires significant investments in hardware and software.
5	Cybersecurity Concerns	I4.0 technologies increase the risk of cyber-attacks and the need for strong cybersecurity measures.
6	Resistance to Change	Employees may resist shifting from traditional practices, creating challenges in adopting and implementing new technologies.
7	Complexity	The intricacy of I4.0 technologies can pose significant challenges for some organizations.
8	Standardization	There is a lack of standardization and interoperability across different systems and platforms.
9	Technology Dependence	Dependence on technology and potential for system failures is a significant issue.
10	Privacy Concerns	Concerns about privacy and security can limit access to data and information.
11	Property rights	Inadequate legal and regulatory frameworks for protecting intellectual property rights pose challenges for adopting I4.0.
12	Reorganization	Automation may replace tasks traditionally performed by humans, reshaping roles within organizations.
13	Infrastructure Challenges	Inadequate infrastructure and connectivity in certain regions, hindering the widespread adoption of I4.0.
14	Limited Talent Pool	A shortage of professionals with expertise in I4.0 makes it difficult for companies to find qualified talent.
15	Integration with Legacy Systems	Integrating new I4.0 technologies with outdated legacy systems remains a significant challenge in the supply chain.

#### 4.1.3 Opportunities (O)

The implementation of I4.0 in the supply chain can also create significant opportunities. To identify these opportunities, we first pinpointed potential new services enabled by I4.0 technologies. We then assessed how these opportunities align with the overall strategic objectives.

Table 5 lists 15 opportunities that I4.0 can offer to the supply chain of the Iranian steel industry.

**Table 5:** Opportunities presented by I4.0.

Opportunities		
1	Sustainable development	I4.0 can significantly contribute to sustainable development by improving the efficiency, productivity, and flexibility of industries.
2	Eco-sustainable production	I4.0 can greatly impact eco-sustainable production by increasing the efficiency, productivity, and flexibility of industries.
3	Removing Barriers	I4.0 will help remove obstacles between investors and markets.
4	Waste Reduction	Improved efficiency from I4.0 will lead to less waste production.
5	Energy Conservation	Increased efficiency due to I4.0 will reduce energy consumption.
6	Improved lead Times	Enhanced connectivity and faster information flow will result in shorter lead times.
7	New Business Models	I4.0 provides opportunities for organizations to develop new business models and revenue streams.
8	Improved Customer Service	I4.0 technologies enhance customer service through real-time monitoring and tracking of goods, enabling quick responses to customer needs.
9	Increased Competitive Advantage	Organizations that adopt I4.0 technologies gain a competitive edge over those that do not.
10	New Products and Services	Data-driven insights enable the development of new products and services, leveraging I4.0 technologies.
11	New Revenue Streams	Exploring data monetization helps organizations identify new revenue streams through I4.0.
12	New Partnerships	Adoption of I4.0 technologies fosters new partnerships and collaborations across the supply chain.
13	Improved resilience	Predictive analytics enhance supply chain resilience and risk management, helping organizations anticipate and mitigate potential disruptions.
14	New Skills and Capabilities	Adopting new technologies fosters the development of new skills and capabilities in the workforce, essential for successful I4.0 implementation.
15	New Job Opportunities	I4.0 creates new job opportunities and employment possibilities.

#### 4.1.4 Threats (T)

Finally, the implementation of I4.0 in the supply chain can also introduce various threats. To identify these threats, we analyzed how the adoption of I4.0 technologies might generate new challenges or risks. Table 6 lists 15 potential threats that I4.0 could pose to the supply chain of the Iranian steel industry.

**Table 6:** Treats presented by I4.0.

Treats		
1	Job Losses	Advent of I4.0, with automation and digitization, is causing job losses, particularly among low-skilled and low-wage workers, leading to social tensions and negative attitudes toward the new industrial revolution.
2	Data Security	In the I4.0 era, data and knowledge are the most valuable assets, making the protection and security of information a critical concern.
3	Cybersecurity Concerns	Cyber threats, such as terrorism, hacking, and cybercrime, are major impediments to the widespread adoption of I4.0.
4	Privacy Concerns	There are concerns about the privacy implications of connecting all devices through IoT and public mistrust of such technology.
5	Implementation Challenges	There is a lack of practical frameworks for implementing I4.0 across various industries.
6	Knowledge Management	A deficiency in appropriate knowledge management systems and platforms can reduce the potential of I4.0.
7	Data Processing Needs	Efficient algorithms are needed to process the massive amounts of data generated by I4.0 technologies.
8	Economic Uncertainty	Economic uncertainty and global instability may impact investments in I4.0 technologies.
9	Regulation and Standardization	Need for regulation and standardization of I4.0 technologies may pose challenges for organizations.
10	Talent Shortage	Talent shortage in I4.0 is a growing concern, especially in regions like Iran, where many skilled professionals emigrate for better opportunities, making it challenging for organizations to find and retain the right talent, particularly in AI, data analytics, and automation.
11	Competitive Adoption of I4.0	There is tough competition from other companies and industries also adopting I4.0 technologies.
12	Technological Risk	Risk of obsolescence and outdated technology is significant.
13	Inequality in Access	Potential for unequal access to technology and market opportunities may result in further market concentration, reduced competition, lack of innovation, and greater economic inequality.
14	Supply Chain Balancing	Balancing the need for efficiency with ethical and sustainable supply chain practices can be challenging.
15	Regulatory Uncertainty	Uncertainty around regulatory and legal frameworks for I4.0 may pose potential hurdles that slow adoption.

### 4.2 Determining SWOT factors using fuzzy Delphi

Group decision-making often involves managing uncertainty and ambiguity. The fuzzy Delphi method offers a robust framework for addressing these challenges by allowing experts to express their opinions using fuzzy numbers and aggregating the information through fuzzy set theory.

**Step 1:** Problem definition and expert selection.

The first step in the fuzzy Delphi method process is to clearly define the problem and select a group of experts in the relevant field. For this study, 12 experts were chosen using a purposive-judgmental method. These experts were selected based on their extensive experience of at least ten years in the steel industry and their comprehensive understanding of I4.0 concepts.

**Step 2:** Expert opinion elicitation.

In this stage, experts were asked to provide their opinions on the dimensions of a specially designed questionnaire using fuzzy numbers (see supplementary materials). A 5-point Likert scale, detailed in Table 7, was employed for this purpose:

**Table 7:** 5-point Likert scale.

Verbal Expressions	Very Important	Important	Relatively Important	Unimportant	Very Unimportant
Fuzzy Numbers	(0.75, 1.00, 1.00)	(0.5, 0.75, 1.00)	(0.25, 0.5, 0.75)	(0.00, 0.25, 0.5)	(0.00, 0.00, 0.25)

**Step 3:** Expert opinion aggregation.

The experts’ opinions were aggregated into a fuzzy set representing an overall expert opinion. This was done using the theory of fuzzy sets, which allows for the combination of fuzzy numbers into a cohesive representation of expert opinions.

Based on the experts’ opinions, the fuzzy value of each question is calculated. For this purpose, it is assumed that the fuzzy value of the *j*th question is represented as $\tilde{A_{j}}=\left(L_{j},M_{j},U_{j}\right)$ where *L*_*j*_ is the lower bound, *M*_*j*_ is the middle value, and *U*_*j*_ is the upper bound of this fuzzy number. Therefore, we have

(1) $$L_{j}=\min\left(x_{ij}\right)\;\left(i=1,2,\ldots ,n;\;j=1,2,\ldots ,m\right)\text{,}$$

(2) $$M_{j}={\left(\prod_{i=1}^{nm}x_{ij}\right)}^{\frac{1}{n}}\;\left(i=1,2,\ldots ,n;\;j=1,2,\ldots ,m\right)\text{,}$$

(3) $$U_{j}=\max\left(x_{ij}\right)\;\left(i=1,2,\ldots ,n;\;j=1,2,\ldots ,m\right)\text{,}$$

where $x_{ij}$ stands for the value assigned by the *i*th expert to the *j*th question.

After calculating the fuzzy value for each of the research questions, these values must be defuzzified to facilitate judgment and comparison. For this purpose, Eq. (4) calculates the defuzzified value for the *j*th question:

(4) $$S_{j}=\frac{L_{j}+2M_{j}+U_{j}}{4}\;\left(j=1,2,\ldots ,m\right)\text{.}$$

After calculating the precise or defuzzified value for each question, their significance needs to be assessed. For this purpose, it is common to use a threshold value (*r*). In this research, following the Pareto principle, a threshold of 0.8 has been considered. Based on this value, two scenarios arise:

If *S*_*j*_ ≥ *r*, the *j*th question is of high importance,

If *S*_*j*_ < *r*, the *j*th question is of low importance and, due to its lower significance, it can be eliminated.

**Step 4:** Feedback and refinement.

The experts provided feedback on the aggregation of their opinions and were asked to revise their opinions based on the feedback. This process was repeated until consensus was reached or a sufficient level of agreement was achieved (two stages).

**Step 5:** Conclusion.

Based on the aggregation of experts’ opinions using the fuzzy Delphi method, the 6 cases that received the highest scores were selected for each SWOT criterion. The scores are described in Table 8.

**Table 8:** Final results of the fuzzy Delphi method.

Strengths		Weaknesses	
Criterion	Score	Criterion	Score
1. Compatibility	0.75	1. Operator Training	0.75
**2. Decentralization**	**0.80**	**2. Upskilling**	**0.88**
**3. Real-time responsiveness**	**0.86**	**3. Data sharing**	**0.86**
4. Modularity	0.79	**4. High Implementation Costs**	**0.89**
5. Service orientation	0.79	5. Cybersecurity Concerns	0.80
6. Efficiency improvement	0.76	**6. Resistance to Change**	**0.88**
**7. Productivity improvement**	**0.84**	7. Complexity	0.81
**8. Flexibility**	**0.85**	8. Standardization	0.82
9. Customer satisfaction	0.70	9. Technology Dependence	0.78
10. Improved Data Management	0.68	**10. Privacy Concerns**	**0.83**
**11. Real-Time Monitoring**	**0.85**	11. Property rights	0.81
12. visibility and traceability	0.65	12. Reorganization	0.79
**13. Decision-Making**	**0.86**	**13. Infrastructure Concerns**	**0.84**
14. Sustainability improvement	0.76	14. Limited Talent Pool	0.77
15. Supply chain security	0.72	15. Integration with Legacy Systems	0.77
**Opportunities**		**Treats**	
**Criterion**	**Score**	**Criterion**	**Score**
1. Sustainable development	0.79	1. Job losses	0.78
2. Eco-sustainable production	0.79	**2. Data security**	**0.82**
**3. Removing Barriers**	**0.87**	3. Cybersecurity concerns	0.78
4. Waste reduction	0.79	4. Privacy concerns	0.76
5. Energy conservation	0.83	**5. Implementation challenges**	**0.87**
**6. Improved lead times**	**0.88**	**6. Knowledge management issues**	**0.83**
7. New business models	0.80	7. Data processing needs	0.78
**8. Improved Customer Service**	**0.86**	**8. Economic Uncertainty**	**0.86**
**9. Increased Competitive Advantage**	**0.86**	9. Regulation and Standardization	0.78
10. New products and services	0.77	**10. Talent Shortage**	**0.84**
**11. New revenue streams**	**0.88**	11. Competitive Adoption	0.78
**12. New partnerships**	**0.84**	12. Technological Risk	0.68
13. Improved resilience	0.77	13. Inequality in Access	0.76
14. New skills and capabilities	0.67	**14. Supply Chain Balancing**	**0.81**
15. New job opportunities	0.61	15. Regulatory Uncertainty	0.76

### 4.3 SWOT policies

The SWOT analysis includes four types of strategic options or policies that the supply chain can develop based on the findings of the analysis. These options, known as SO, SW, TO, and TW, are formulated by considering both internal and external factors identified in the analysis. They are designed to assist the supply chain in leveraging their strengths, addressing their weaknesses, taking advantage of opportunities, and mitigating threats.

• **SO: Strengths-Opportunities policy**

This policy is based on the supply chain's strengths and opportunities. It involves developing strategies that leverage the supply chain's strengths to take advantage of opportunities.

• **ST: Strengths-Threats policy**

This policy involves using the strengths of the supply chain to counteract or minimize the impact of threats.

• **WO: Weaknesses-Opportunities policy**

This policy involves using opportunities to address weaknesses in the supply chain.

• **WT: Weaknesses-Threats policy**

This policy involves minimizing weaknesses and avoiding threats.

**Fig. 8:**
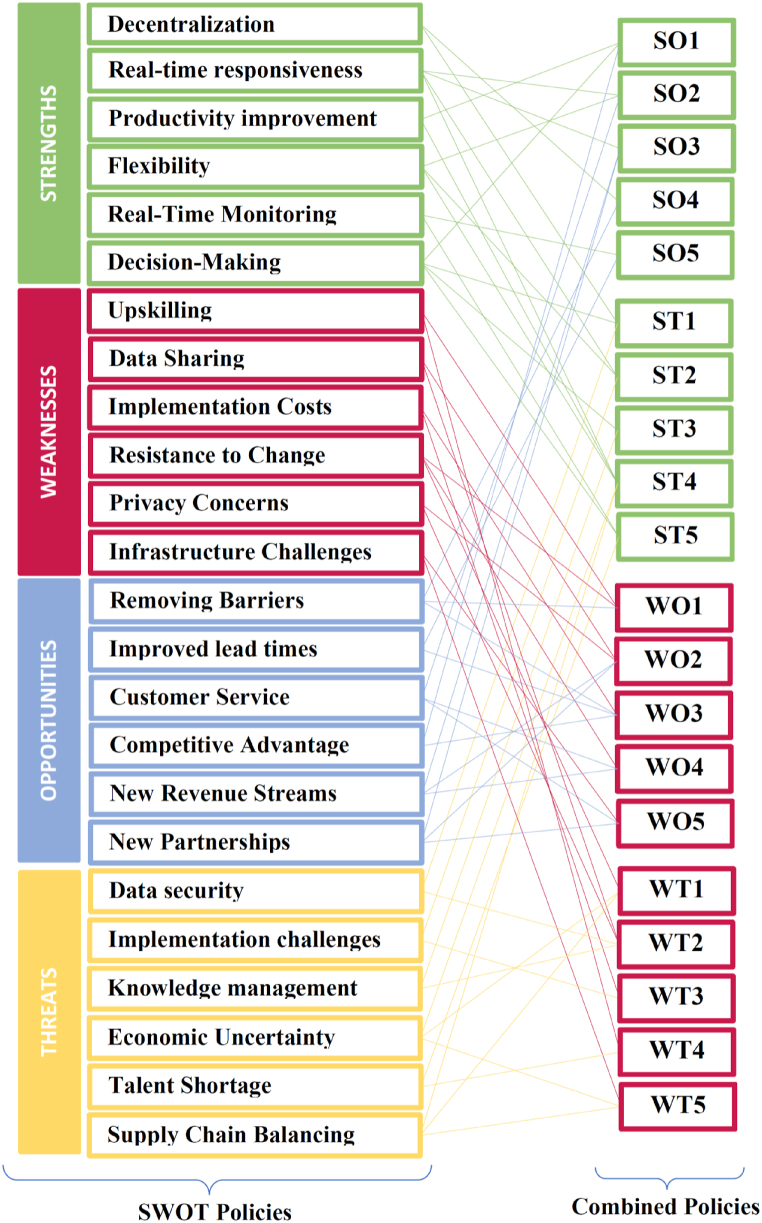
SWOT policies and combined policies.

In summary, the SO, ST, WO, and WT policies are strategies that can be developed based on the results of a SWOT analysis to help the supply chain take advantage of opportunities, minimize weaknesses, and address threats in the market. Based on the selected SWOT factors in the previous section and consultation with the experts, significant policies were formulated for the selected factors. These policies are shown in Table 9. Furthermore, Fig. 8 illustrates the process of creating combined policies from SWOT policies.

**Table 9:** SO, SW, to, and TW policies.

SO	1. Invest in I4.0 technologies to enhance decision-making, increase productivity, and gain a competitive advantage.
	2. Leverage I4.0 to improve flexibility, real-time responsiveness, and customer service.
	3. Create new revenue streams through data monetization, increased productivity, and exploring new partnerships across the supply chain.
	4. Use I4.0 to decentralize and remove barriers between investors and markets.
	5. Utilize I4.0 for real-time monitoring to improve lead times.
**ST**	1. Enable decentralized decision-making and implement robust data security measures to protect valuable information.
	2. Improve real-time responsiveness and flexibility by developing practical frameworks for implementing I4.0 technologies.
	3. Develop knowledge management systems and platforms to ensure effective data utilization and support data-driven decision-making.
	4. Improve real-time responsiveness and flexibility to mitigate economic uncertainty and global economic instability, while balancing the supply chain to ensure productivity.
	5. Implement training and education programs to address the talent shortage in I4.0 and enable effective decision-making and real-time monitoring.
**WO**	1. Develop upskilling programs to ensure employees have the necessary digital skills, seizing the opportunity for a competitive advantage while addressing the weakness of upskilling.
	2. Address data privacy concerns to increase access to data and information, leveraging the opportunities for revenue streams and new partnerships.
	3. Invest in I4.0 technologies to remove barriers and gain a competitive advantage, reducing lead time and implementation costs, and seizing the opportunity for revenue streams.
	4. Foster a culture of innovation to encourage employees to embrace change and adopt I4.0 technologies, utilizing the opportunities for revenue streams and customer service.
	5. Address infrastructure issues to facilitate the adoption of I4.0 technologies, capitalizing on the opportunity for new partnerships and improving customer service.
**WT**	1. Develop policies that balance the need for efficiency with ethical and sustainable supply chain practices, minimizing the weakness of resistance to change and avoiding the threat of economic uncertainty and global instability.
	2. Develop policies that address data security concerns to mitigate risks, minimize the weakness of data sharing, and avoid the threat of knowledge management challenges.
	3. Develop policies that provide practical frameworks for successful digital transformation, minimizing the weakness of implementation costs and avoiding the threat of implementation challenges.
	4. Develop policies that address talent shortage through investment in education and training programs, minimizing the weakness of upskilling and avoiding the threat of talent shortage.
	5. Develop policies that account for economic uncertainty and global instability in supply chain strategies, minimizing the weakness of infrastructure and avoiding the threat of supply chain balancing.

### 4.4 Determination of the optimal combination of strategies using game theory

Game theory is a field within mathematics that focuses on examining decision-making in scenarios where the outcomes are impacted by the choices made by several individuals or groups. It offers a structure for analyzing strategic interactions and forecasting the actions of rational actors.

Suppose we have a two-player game with Players 1 and 2. The payoff for Player 1 for choosing strategy $s_{1}$ and Player 2 for choosing strategy $s_{2}$ is denoted by $u_{1}\left(s_{1},s_{2}\right)$ and $u_{2}\left(s_{1},s_{2}\right)$, respectively.

A Nash equilibrium is a pair of strategies $u_{1}\left(s_{1}^{*},s_{2}^{*}\right)$ such that no player can unilaterally change their strategy to increase their payoff. In other words, if Player 1 selects $s_{1}^{*}$ and Player 2 selects $s_{2}^{*}$*,* then neither player can improve their payoff by changing their strategy. Formally, a Nash equilibrium $\left(s_{1}^{*},s_{2}^{*}\right)$ satisfies the following conditions:

(5) $$\begin{array}{ll} u_{1}\left(s_{1}^{*},s_{2}^{*}\right)\geq u_{1}\left(s_{1},s_{2}^{*}\right) & \forall s_{1}\text{,}\\ u_{2}\left(s_{1}^{*},s_{2}^{*}\right)\geq u_{2}\left(s_{1}^{*},s_{2}\right) & \forall s_{2}.\; \end{array}$$

In other words, these conditions say that neither player can increase their payoff by unilaterally changing their strategy, given that the other player is using their Nash equilibrium strategy.

In the case of mixed strategies, where players choose strategies with some probability, the Nash equilibrium condition becomes:

(6) $$\begin{array}{ll} u_{1}\left(s_{1}^{*},s_{2}^{*}\right)\geq u_{1}\left(s_{1},s_{2}^{*}\right)\;\forall s_{1}\text{,} & \\ u_{2}\left(s_{1}^{*},s_{2}^{*}\right)\geq u_{2}\left(s_{1}^{*},s_{2}\right)\;\forall s_{2}\text{,} & \\ \sum_{s_{1}}\;p_{1}\left(s_{1}\right)u_{1}\left(s_{1},s_{2}^{*}\right)\geq \sum_{s_{1}}\;p_{1}\left(s_{1}\right)u_{1}\left(s_{1},s_{2}\right) & \forall s_{2}\text{,}\\ \sum_{s_{2}}\;p_{2}\left(s_{2}\right)u_{2}\left(s_{1}^{*},s_{2}\right)\geq \sum_{s_{2}}\;p_{2}\left(s_{2}\right)u_{2}\left(s_{1},s_{2}\right) & \forall s_{1}, \end{array}$$

where $p_{1}$ and $p_{2}$ are the probability distributions over the strategies of Players 1 and 2, respectively. The last two conditions ensure that neither player can increase their expected payoff by changing their mixed strategy, given that the other player is using their Nash equilibrium mixed strategy.

First, we aim to obtain a coalition of the SO and ST policies, as well as a coalition of the WO and WT policies using game theory. Then, we create an overall coalition of the SO, ST, WO, and WT policies. We created a matrix to form a coalition of the SO and ST policies. The SO/ST table shows the possible combined strategies that can be created by combining identified strengths and opportunities (SO) and strengths and threats (ST). We asked research experts to register the appropriate option for policy coalition with each other. A 5-degree Likert spectrum was used for scoring. The results of the scores are shown in Table 10. It must be noted that the coding of the suggested Game theoretic method was performed in Python software.

**Table 10:** Results of the SO/ST scores.

ST/SO	SO1	SO2	SO3	SO4	SO5
**ST1**	0.92	0.85	0.82	0.85	0.89
**ST2**	0.89	0.82	0.85	0.89	0.92
**ST3**	0.85	0.80	0.77	0.82	0.85
**ST4**	0.85	0.82	0.77	0.85	0.85
**ST5**	0.82	0.84	0.77	0.85	0.82

Nash equilibrium is a concept in game theory that refers to a scenario where every player in a game chooses their optimal strategy, taking into consideration the choices made by other players. To apply this concept to the SO/ST table, each row can be viewed as a player and each column as a strategy. The table displays payoffs for each player when they choose a particular strategy.

In the code, a payoff matrix was created from the SO/ST table, with rows representing SO strategies and columns representing ST strategies. The Nashpy library was then utilized to determine the Nash equilibrium of the payoff matrix. The Nash equilibrium assists in identifying the best SO/ST hybrid strategy as a response to the choices made by other players in the game.

By identifying the Nash equilibrium, the best SO/ST hybrid strategy that maximizes payoffs for each player given the other player's choices can be determined. The code sorts the Nash equilibrium values in descending order and selects the SO/ST hybrid strategy with the highest Nash equilibrium value as the best strategy.

The output of the code provides a table of Nash Equilibrium values (see Table 11) for each combination of SO and ST.

**Table 11:** Nash Equilibrium values for each combination of SO/ST.

Nash Equilibrium Values Table	1	2	3	4	5	Nash equilibrium value
SO	1.5	1.5	1	1	1	0.835
ST	1	1.5	1.5	1	1	−0.835

The Nash Equilibrium value is a measure of how well a given strategy combination will perform against an opponent who is also playing optimally. In this case, the Nash Equilibrium value is a two-element array, which represents the expected payoffs for each player in the game. The best SO/ST hybrid strategy is the one with the highest Nash Equilibrium value. In this case, the selected SO strategy is a combination of SO1, and SO2 with equal weights, and the selected ST strategy is a combination of ST2, and ST3, with equal weights, which has a Nash Equilibrium value of [0.835–0.835].

Similarly, we used game theory to create a coalition of WO and WT policies.

The WO/WT table displays potential hybrid strategies that can be formed by combining identified weaknesses and opportunities (WO) as well as weaknesses and threats (WT) policies. Similar to the SO/ST table, the WO/WT table is populated with fuzzy numbers assigned by research experts based on their analysis and judgment (see Table 12).

**Table 12:** Results of the WO/WT scores.

WO/WT	WO1	WO2	WO3	WO4	WO5
**WT1**	0.77	0.85	0.82	0.65	0.60
**WT2**	0.89	0.85	0.89	0.82	0.82
**WT3**	0.73	0.85	0.70	0.68	0.65
**WT4**	0.60	0.77	0.85	0.70	0.65
**WT5**	0.85	0.89	0.85	0.78	0.73

The output of the code provides a table of Nash Equilibrium values for each combination of WO and WT (see Table 13).

**Table 13:** Nash Equilibrium values for each combination of WO/WT.

Nash Equilibrium Values Table	1	2	3	4	5	Nash equilibrium value
WO	1	2	1	1	1	0.82
WT	1	1	1	2	2	−0.82

In this case, the selected WO strategy is WO2, and the selected WT strategy is a combination of WT4, and WT5, with equal weights, which has a Nash Equilibrium value of [0.82–0.82].

Therefore, the selected strategies according to game theory are given in Table 14 After selecting the strategies, the subsequent step involves carrying out a gap analysis.

**Table 14:** Selected strategies according to game theory.

SO1	Invest in I4.0 technologies to enhance decision-making, increase productivity, and gain a competitive advantage.
SO2	Leverage I4.0 to improve flexibility, real-time responsiveness, and customer service.
ST2	Improve real-time responsiveness and flexibility by developing practical frameworks for implementing I4.0 technologies.
ST3	Develop knowledge management systems and platforms to ensure effective data utilization and support data-driven decision-making.
WO2	Address data privacy concerns to increase access to data and information, leveraging the opportunities for revenue streams and new partnerships.
WT4	Develop policies that address talent shortage through investment in education and training programs, minimizing the weakness of upskilling and avoiding the threat of talent shortage.
WT5	Develop policies that account for economic uncertainty and global instability in supply chain strategies, minimizing the weakness of infrastructure and avoiding the threat of supply chain balancing.

### 4.5 Gap analysis

Gap analysis is a vital aspect of performance management and improvement. It assists organizations in pinpointing areas that need improvement, prioritizing initiatives, and assigning resources more effectively. By comparing current performance against desired goals, gap analysis provides a roadmap for bridging the gap and achieving success.

**Fig. 9:**
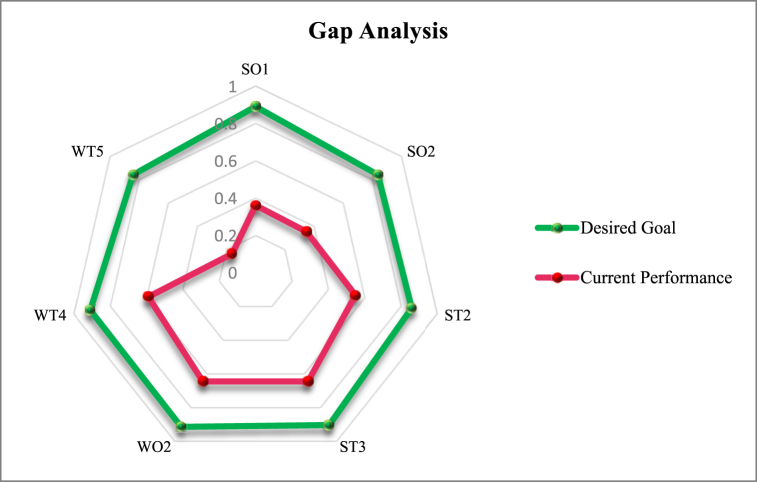
Desired goal vs. current performance based on the gap analysis.

According to the aggregation of experts’ opinions using the fuzzy Delphi method, the current performance and desired goals were determined for each strategy. The scores are shown in Table 15 and Fig. 9.

**Table 15:** Gap analysis results.

Strategy	Current performance	Desired goal
SO1	0.36	0.89
SO2	0.35	0.84
ST2	0.55	0.86
ST3	0.65	0.91
WO2	0.65	0.92
WT4	0.59	0.91
WT5	0.16	0.84

### 4.6 Discussion

This research offers a comprehensive roadmap for leveraging strengths and addressing weaknesses, enabling organizations to capitalize on opportunities and mitigate threats effectively. The results from our gap analysis were pivotal in highlighting areas requiring improvement. By assessing the differences between current performance and desired objectives for each strategy, organizations can concentrate their efforts on areas with the greatest potential for enhancement.

Our study also emphasized the crucial role of a robust infrastructure and a well-balanced supply chain. These elements are essential for the successful integration of I4.0 technologies, contributing to both performance optimization and risk mitigation.

We emphasized the importance of a well-informed approach to I4.0 adoption. Based on our research, organizations are encouraged to consider the integration of identified SWOT strategies. Furthermore, prioritization should be informed by the gap analysis, ensuring that resources are allocated judiciously to address the most significant disparities between current performance and desired objectives.

#### 4.6.1 Key findings and implications

• SWOT Analysis: The identified SWOT factors highlight the critical areas that organizations need to address for successful I4.0 implementation. For example, leveraging strengths (e.g., real-time responsiveness) and addressing weaknesses (e.g., data privacy concerns) are essential.
• Gap Analysis: The gap analysis underscores the significant disparities between the current state of the Iranian steel supply chain and the desired state of I4.0. This highlights the need for targeted interventions to bridge the gap.
• Infrastructure and Supply Chain Balancing: The research emphasizes the crucial role of a robust infrastructure and balanced supply chain in facilitating I4.0 adoption. Organizations must invest in these areas to ensure smooth operations and minimize risks.

#### 4.6.2 Comparison to existing literature

Previous research had investigated the factors influencing I4.0 on the supply chain, but none had provided a comprehensive review of these factors. By studying and summarizing over 50 previous articles, our research extends previous studies by outlining key factors influencing I4.0 on the supply chain and hybrid study of SWOT strategies in the context of I4.0 and gap analysis of a big supply chain. Overall, this study contributes to the existing literature by providing a comprehensive analysis of I4.0 implementation in the steel supply chain. The findings offer practical guidance for organizations seeking to optimize their I4.0 adoption efforts and contribute to the ongoing development of this emerging field.

#### 4.6.3 Managerial and practical implications

• Strategic Planning: Organizations should prioritize the identified SWOT factors in their strategic planning processes and allocate resources accordingly.
• Organizational Change Management: Implementing I4.0 requires significant organizational change. Organizations should develop effective change management strategies to overcome resistance and ensure successful adoption.
• Talent Management: The transition to I4.0 requires a skilled workforce. Organizations should invest in training and development programs to equip their employees with the necessary skills.
• Technology Investment: The identified SWOT factors can inform decisions about which I4.0 technologies to prioritize for investment.
• Risk Management: Organizations should develop strategies to mitigate the risks associated with I4.0 implementation, such as cybersecurity threats and supply chain disruptions.

## 5 Conclusion and outlook

This research aimed to identify the most critical factors influencing the implementation of I4.0 in the supply chain of large industries, develop and evaluate the most significant policies for implementing these factors, and analyze the gap between the current state of the Iranian steel supply chain and the desired state of I4.0. Therefore, after SWOT analysis and selecting the optimal combination of strategies using game theory, the current situation gap of the Iranian steel supply chain was analyzed with the desired state (objectives) of I4.0. The selected policies using game theory were as follows.

I. Invest in I4.0 technologies to enhance decision-making, increase productivity, and gain a competitive advantage.
II. Leverage I4.0 to improve flexibility, real-time responsiveness, and customer service.
III. Improve real-time responsiveness and flexibility by developing practical frameworks for implementing I4.0 technologies.
IV. Develop knowledge management systems and platforms to ensure effective data utilization and support data-driven decision-making.
V. Address data privacy concerns to increase access to data and information, leveraging the opportunities for revenue streams and new partnerships.
VI. Develop policies that address talent shortage through investment in education and training programs, minimizing the weakness of upskilling and avoiding the threat of talent shortage.
VII. Develop policies that account for economic uncertainty and global instability in supply chain strategies, minimizing the weakness of infrastructure and avoiding the threat of supply chain balancing.

The selection of these policies may indicate the following.

i. Steel experts anticipate that investing in I4.0 technologies can optimize the supply chain decision-making process.
ii. Real-time responsiveness to customers is a crucial issue that I4.0 can address effectively.
iii. In the field of I4.0, slogans alone are not sufficient. A practical framework for implementing these technologies in the supply chain is essential.
iv. Effective knowledge management is a prerequisite for utilizing data, including I4.0 technologies.
v. Concerns about data privacy must be addressed proactively, and data security is of utmost importance.
vi. Investment in education can provide sufficient talent in the field of current technologies.
vii. Effective strategies should be developed to address the current economic uncertainties in Iran and global instabilities.

### 5.1 Directions for future research

In accordance with the conducted comprehensive analysis, limitations, and recommendations for future research include.

➢ **Data Availability and Quality:** One of the primary limitations of our study is data availability. Future research should strive to access comprehensive datasets to provide more accurate insights. Collaborations with industry stakeholders and governmental bodies could facilitate data sharing and collection.
➢ **Generalizability:** Our research primarily focused on the Iranian steel supply chain. However, the effectiveness of I4.0 adoption may vary across different nations and industries. Future studies should aim to replicate our research in diverse contexts to enhance generalizability.
➢ **Long-term Impact Assessment:** Considering the dynamic nature of technology, it is important to explore the long-term effects of I4.0 implementation. Future research can conduct longitudinal studies to assess how these technologies influence supply chains over extended periods, offering a more comprehensive view of their impact.
➢ **Ethical and Societal Implications:** I4.0 adoption introduces ethical and societal concerns, such as job displacement and data privacy. Future research can delve into these dimensions, guiding how to address these issues effectively and responsibly.
➢ **Comparative Studies:** To enhance our understanding of I4.0's influence, future research can conduct comparative studies across industries and countries. By analyzing similarities and differences, these studies can offer valuable insights into the nuances of I4.0 implementation in various contexts.
➢ **Studying the Effectiveness of I4.0 Implementation Across Diverse Sectors:** While our research has focused on the Iranian steel supply chain, there's room for future studies to explore the impact of I4.0 implementation in various industries. Case studies and performance evaluations can provide valuable insights into how different sectors leverage I4.0 to enhance their supply chain operations. This would allow for broader generalizations and insights that could be applied to different industries.
➢ **Examining the Environmental Implications of I4.0 Implementation:** As I4.0 technologies continue to proliferate, their effects on the environment are a subject of growing concern. Future research must explore the environmental aspects of I4.0 adoption. This includes assessing the ecological footprint of these technologies and developing strategies for more sustainable and environmentally friendly implementation.
➢ **New Approaches:** There are several advanced and emerging methodologies that warrant further exploration in future research. These include sophisticated techniques and theoretical frameworks that have shown promise in various applications. Stochastic optimal control [38] and cooperative games under bubble uncertainty [39] are known as powerful tools for decision-making in environments characterized by uncertainty and dynamic changes. Moreover, new contributions in uncertainty and hybrid models such as Infinite Kernel Learning (IKL) [40], Conic Multivariate Adaptive Regression Splines (CMARS) [41], Robust CMARS (RCMARS) [42], Robust MARS (RMARS) [43], and Generalized Partial Linear Model (GPLM) [44] offer robust approaches for modeling complex systems with multiple interacting components. Incorporating these advanced methodologies could open new avenues for research, particularly in areas that require a high degree of precision and adaptability.

## CRediT authorship contribution statement

**Sima Motallebi:** Writing – original draft, Software, Methodology, Data curation. **Mostafa Zandieh:** Writing – review & editing, Visualization, Supervision, Investigation, Conceptualization. **Akbar Alem Tabriz:** Writing – review & editing, Validation, Formal analysis, Data curation. **Erfan Babaee Tirkolaee:** Writing – review & editing, Validation, Investigation, Formal analysis.

## Ethical statement

This research involved the collection of expert opinions from some managers working in the Iranian steel supply chain. Participants were selected through a purposive and judgmental sampling method based on their extensive experience (at least 10 years) in the steel industry and their comprehensive understanding of I4.0 concepts.

Informed consent was obtained from all participants prior to their involvement in the study. The research was conducted in accordance with ethical guidelines and regulations, and no human subjects were directly involved in experiments or clinical trials.

## Funding

This research did not receive any specific funding.

## Declaration of competing interest

The authors declare that they have no known competing financial interests or personal relationships that could have appeared to influence the work reported in this paper.
