# Supplementary material for: Assessing the industry 4.0 strategies for a steel supply chain: SWOT, game theory, and gap analysis
Source: Heliyon. 2024 Dec 18;11(1):e41374. doi: 10.1016/j.heliyon.2024.e41374 (PMC11730224; doi:10.1016/j.heliyon.2024.e41374)
Supplement: Multimedia component 1 [file mmc1.docx]

***Supplementary Materials***

1. **Questionnaire for determining SWOT factors of Industry 4.o in supply chain**

Dear valued respondent,

Respectfully, the questionnaire at hand has been designed for selecting the significant factors in the SWOT analysis of the steel supply chain. We appreciate your valuable time dedicated to completing this questionnaire and kindly request you to provide answers to the following questions.

With sincere gratitude

| Demographic Information: | |
| --- | --- |
|  | Age: |
|  | Education Level: |
|  | Years of Experience in the Steel Industry: |
|  | Level of familiarity with the concept of Industry 4.0: |

Please select the level of importance of the following factors in relation to the employment of Industry 4.0 in the steel supply chain among the options provided.

| Very Unimportant | Unimportant | Relatively Important | Important | Very Important | **Strengths:** |
| --- | --- | --- | --- | --- | --- |
|  |  |  |  |  | 1. Compatibility: The ability of different industries to share or utilize each other's machinery and equipment that have similar functions is improved. |
|  |  |  |  |  | 2. Decentralization: Industry 4.0 enhances the capability of machines, personnel, and small businesses to make quick and data-driven decisions. |
|  |  |  |  |  | 3. Improved real-time responsiveness: The speed of response is increased, allowing machines to adapt to customer requirements with greater speed and efficiency. |
|  |  |  |  |  | 4. Modularity: Industry 4.0 allows the production process to adjust easily to changes in product design or seasonal fluctuations. |
|  |  |  |  |  | 5. Service orientation: Companies, people, and CPS are able to interact with each other through the Internet of Things, leading to better value for supply chain. |
|  |  |  |  |  | 6. Efficiency improvement: Industry 4.0 technologies like automation, robotics and Internet of Things (IoT) increase the efficiency of supply chain processes. Industry 4.0 also enhances efficiency, reducing the use of energy and raw materials. |
|  |  |  |  |  | 7. Productivity improvement: The integration of smart technologies in Industry 4.0 leads to increased productivity and Reduced costs through optimization and streamlining of processes. |
|  |  |  |  |  | 8. Flexibility: Industry 4.0 technologies provide greater flexibility in terms of production and supply chain processes, enabling organizations to respond quickly to changes in demand. |
|  |  |  |  |  | 9. Higher customer satisfaction: The integration of customers into the production process through a network in Industry 4.0 and personalized and timely delivery of goods. leads to greater customer satisfaction. |
|  |  |  |  |  | 10. Improved Data Management: The integration of Big Data and advanced analytics in the supply chain enhances decision making and improves data management. |
|  |  |  |  |  | 11. Real-Time Monitoring: The use of sensors and connected devices enables real-time monitoring and tracking of goods, reducing the risk of lost or damaged goods. |
|  |  |  |  |  | 12. visibility and traceability. Industry 4.0 leverages technologies such as IoT, Big Data, and AI to improve supply chain visibility and traceability. This can be achieved through real-time monitoring and analysis of supply chain data, enabling quick identification and resolution of potential issues. |
|  |  |  |  |  | 13. Decision-Making: Industry 4.0 Enhances decision-making through data-driven insights. |
|  |  |  |  |  | 14. Sustainability improvement: Industry 4.0 Improves sustainability through resource optimization and reduced waste. |
|  |  |  |  |  | 15. Supply chain security: Industry 4.0 can improve supply chain security through the use of technologies such as blockchain, IoT devices, and advanced data analytics. These technologies provide secure and transparent methods of tracking and monitoring supply chain activities, reducing the risk of fraud, counterfeiting and theft. |

| Very Unimportant | Unimportant | Relatively Important | Important | Very Important | **Weaknesses:** |
| --- | --- | --- | --- | --- | --- |
|  |  |  |  |  | 1. Operator Training: Training of operators and improvement of their skills to handle digital tasks. |
|  |  |  |  |  | 2. Upskilling: Providing operators with new skills and transforming the workforce to allow for successful digital task management. |
|  |  |  |  |  | 3. Data sharing: Sharing of data and information among competing industries. |
|  |  |  |  |  | 4. High Implementation Costs: Implementing Industry 4.0 technologies can be expensive and requires significant investments in hardware and software. |
|  |  |  |  |  | 5. Cybersecurity Concerns: Industry 4.0 technologies increase the risk of cyber-attacks and the need for strong cybersecurity measures. |
|  |  |  |  |  | 6. Resistance to Change: Employees may resist changes to the traditional way of doing things, leading to difficulties in adoption and implementation. |
|  |  |  |  |  | 7. Complexity: The complexity of Industry 4.0 technologies may present a challenge for some organizations. |
|  |  |  |  |  | 8. Standardization: Lack of standardization and interoperability across different systems and platforms. |
|  |  |  |  |  | 9. Dependence: Dependence on technology and a potential for system failures. |
|  |  |  |  |  | 10. Privacy: Limited access to data and information due to privacy and security concerns. |
|  |  |  |  |  | 11. Property rights: Inadequate legal and regulatory frameworks for the protection of intellectual property rights. |
|  |  |  |  |  | 12. Reorganization: Some tasks previously done by humans may now be automated. |
|  |  |  |  |  | 13. Infrastructure: Inadequate infrastructure and connectivity in certain regions, hindering the widespread adoption of Industry 4.0. |
|  |  |  |  |  | 14. Limited Talent Pool: A shortage of skilled professionals with the necessary expertise in Industry 4.0 technologies, making it difficult for organizations to find the right talent. |
|  |  |  |  |  | 15. Integration with Legacy Systems: The challenge of integrating new Industry 4.0 technologies with older, legacy systems in the supply chain. |

| Very Unimportant | Unimportant | Relatively Important | Important | Very Important | **Opportunities:** |
| --- | --- | --- | --- | --- | --- |
|  |  |  |  |  | 1. Sustainable development: Industry 4.0 can greatly contribute to sustainable development through improvements in efficiency, productivity, and flexibility of industries. |
|  |  |  |  |  | 2. Eco-sustainable production: Industry 4.0 can also have a significant impact on eco-sustainable production by increasing efficiency, productivity, and flexibility in industries. |
|  |  |  |  |  | 3. Removing Barriers: Industry 4.0 will aid in removing obstacles that exist between investors and markets. |
|  |  |  |  |  | 4. Waste reduction: As a result of improved efficiency, Industry 4.0 will result in less waste being produced. |
|  |  |  |  |  | 5. Energy conservation: Due to increased efficiency, Industry 4.0 will lead to a reduction in energy usage. |
|  |  |  |  |  | 6. Improved lead times: Better connectivity and fast information flow will result in shorter lead times. |
|  |  |  |  |  | 7. New business models: Industry 4.0 provides opportunities for organizations to develop new business models and revenue streams. |
|  |  |  |  |  | 8. Improved Customer Service: Industry 4.0 technologies improve customer service through real-time monitoring and tracking of goods, enabling organizations to respond quickly to customer needs. |
|  |  |  |  |  | 9. Increased Competitive Advantage: Organizations that adopt Industry 4.0 technologies gain a competitive advantage over those that do not. |
|  |  |  |  |  | 10. New products and services: The development of new products and services through data-driven insights. |
|  |  |  |  |  | 11. New revenue streams: The exploration of new revenue streams through data monetization. |
|  |  |  |  |  | 12. New partnerships: The creation of new partnerships and collaborations across the supply chain. |
|  |  |  |  |  | 13. Improved resilience: The improvement of supply chain resilience and risk management through the use of predictive analytics. |
|  |  |  |  |  | 14. New skills and capabilities: The development of new skills and capabilities through the adoption of new technologies. |
|  |  |  |  |  | 15. New job opportunities: The creation of new jobs and employment opportunities in the industry 4.0 sector. |

| Very Unimportant | Unimportant | Relatively Important | Important | Very Important | **Threats:** |
| --- | --- | --- | --- | --- | --- |
|  |  |  |  |  | 1. Job losses: The advent of Industry 4.0, marked by the rise of automation and digitization, is causing job losses, particularly among low-skilled and low-wage workers. This is fueling social tensions and negative attitudes towards the new industrial revolution. |
|  |  |  |  |  | 2. Data security: In the Industry 4.0 era, data and knowledge will be the most valuable assets, making the protection and security of information a critical concern. |
|  |  |  |  |  | 3. Cybersecurity concerns: Cyber threats such as terrorism, hacking, and cybercrime are a major impediment to the widespread adoption of Industry 4.0. |
|  |  |  |  |  | 4. Privacy concerns: Concerns about the privacy implications of connecting all devices through the Internet of Things (IoT), and the mistrust of such technology among the public. |
|  |  |  |  |  | 5. Implementation challenges: There is a lack of practical frameworks for implementing Industry 4.0 in various industries. |
|  |  |  |  |  | 6. Knowledge management issues: A deficiency of appropriate knowledge management systems and platforms. |
|  |  |  |  |  | 7. Data processing needs: The need for efficient algorithms to process massive amounts of data generated by Industry 4.0 technologies. |
|  |  |  |  |  | 8. Economic Uncertainty: Economic uncertainty and global economic instability may impact investment in Industry 4.0 technologies. |
|  |  |  |  |  | 9. Regulation and Standardization: The need for regulation and standardization of Industry 4.0 technologies may pose a challenge for organizations. |
|  |  |  |  |  | 10. Talent Shortage: The need for skilled professionals in Industry 4.0 may lead to a talent shortage, making it difficult for organizations to find and retain the right talent. |
|  |  |  |  |  | 11. Competitive Adoption of Industry 4.0: Competition from other companies and industries adopting Industry 4.0 technologies. |
|  |  |  |  |  | 12. Technological Risk: The risk of obsolescence and outdated technology. |
|  |  |  |  |  | 13. Inequality in Access: The potential for unequal access to technology and market opportunities, resulting in further market concentration. |
|  |  |  |  |  | 14. Supply Chain Balancing: The difficulty of balancing the need for efficiency with the importance of ethical and sustainable supply chain practices. |
|  |  |  |  |  | 15. Regulatory Uncertainty: The uncertainty of regulatory and legal frameworks for Industry 4.0 and the potential for regulatory hurdles to slow adoption. |

1. **Questionnaire for GT analysis**

Dear valued respondent,

Respectfully, the questionnaire at hand has been designed for selecting the significant combined policies of SWOT analysis of the steel supply chain. We appreciate your valuable time dedicated to completing this questionnaire and kindly request you to provide answers to the following questions.

With sincere gratitude

First, please read the combined SO and ST policies below.

| **SO** | 1. Invest in Industry 4.0 technologies to enhance decision-making, increase productivity, and gain a competitive advantage. |
| --- | --- |
|  | 1. Leverage Industry 4.0 to improve flexibility, real-time responsiveness, and customer service. |
|  | 1. Create new revenue streams through data monetization, increased productivity, and exploring new partnerships across the supply chain. |
|  | 1. Use Industry 4.0 to decentralize and remove barriers between investors and markets. |
|  | 1. Utilize Industry 4.0 for real-time monitoring to improve lead times. |
| **ST** | 1. Enable decentralized decision-making and implement robust data security measures to protect valuable information. |
|  | 1. Improve real-time responsiveness and flexibility by developing practical frameworks for implementing Industry 4.0 technologies. |
|  | 1. Develop knowledge management systems and platforms to ensure effective data utilization and support data-driven decision-making. |
|  | 1. Improve real-time responsiveness and flexibility to mitigate economic uncertainty and global economic instability, while balancing the supply chain to ensure productivity. |
|  | 1. Implement training and education programs to address the talent shortage in Industry 4.0 and enable effective decision-making and real-time monitoring. |

In the SO/ST matrix, please compare the options and rate their importance to each other according to the verbal expressions table.

| Very Unimportant | Unimportant | Relatively Important | Important | Very Important | **Verbal Expressions** |
| --- | --- | --- | --- | --- | --- |
| e | d | c | b | a | Points |

| SO/ST | SO1 | SO2 | SO3 | SO4 | SO5 |
| --- | --- | --- | --- | --- | --- |
| ST1 |  |  |  |  |  |
| ST2 |  |  |  |  |  |
| ST3 |  |  |  |  |  |
| ST4 |  |  |  |  |  |
| ST5 |  |  |  |  |  |

Please read the combined WO and WT policies below.

| **WO** | 1. Develop upskilling programs to ensure employees have the necessary digital skills, seizing the opportunity for a competitive advantage while addressing the weakness of upskilling. |
| --- | --- |
|  | 1. Address data privacy concerns to increase access to data and information, leveraging the opportunities for revenue streams and new partnerships. |
|  | 1. Invest in Industry 4.0 technologies to remove barriers and gain a competitive advantage, reducing lead time and implementation costs, and seizing the opportunity for revenue streams. |
|  | 1. Foster a culture of innovation to encourage employees to embrace change and adopt Industry 4.0 technologies, utilizing the opportunities for revenue streams and customer service. |
|  | 1. Address infrastructure issues to facilitate the adoption of Industry 4.0 technologies, capitalizing on the opportunity for new partnerships and improving customer service. |
| **WT** | 1. Develop policies that balance the need for efficiency with ethical and sustainable supply chain practices, minimizing the weakness of resistance to change and avoiding the threat of economic uncertainty and global instability. |
|  | 1. Develop policies that address data security concerns to mitigate risks, minimize the weakness of data sharing, and avoid the threat of knowledge management challenges. |
|  | 1. Develop policies that provide practical frameworks for successful digital transformation, minimizing the weakness of implementation costs and avoiding the threat of implementation challenges. |
|  | 1. Develop policies that address talent shortage through investment in education and training programs, minimizing the weakness of upskilling and avoiding the threat of talent shortage. |
|  | 1. Develop policies that account for economic uncertainty and global instability in supply chain strategies, minimizing the weakness of infrastructure and avoiding the threat of supply chain balancing. |

In the WO/WT matrix, please compare the options and rate their importance to each other according to the verbal expressions table.

| Very Unimportant | Unimportant | Relatively Important | Important | Very Important | **Verbal Expressions** |
| --- | --- | --- | --- | --- | --- |
| e | d | c | b | a | Points |

| WO/WT | WO1 | WO2 | WO3 | WO4 | WO5 |
| --- | --- | --- | --- | --- | --- |
| WT1 |  |  |  |  |  |
| WT2 |  |  |  |  |  |
| WT3 |  |  |  |  |  |
| WT4 |  |  |  |  |  |
| WT5 |  |  |  |  |  |

1. **Questionnaire for Gap analysis**

Dear valued respondent,

Respectfully, the questionnaire at hand has been designed for gap analysis between current performance and desired goals of the steel supply chain. We appreciate your valuable time dedicated to completing this questionnaire and kindly request you to provide answers to the following questions.

With sincere gratitude

Please read the SO, ST, WO and WT combined policies in the table and score the current performance of the steel supply chain and the desired performance that you think should exist.

| Desired goal | Current performance | **Strategy** |  |
| --- | --- | --- | --- |
|  |  | Invest in Industry 4.0 technologies to enhance decision-making, increase productivity, and gain a competitive advantage. | SO1 |
|  |  | Leverage Industry 4.0 to improve flexibility, real-time responsiveness, and customer service. | SO2 |
|  |  | Improve real-time responsiveness and flexibility by developing practical frameworks for implementing Industry 4.0 technologies. | ST2 |
|  |  | Develop knowledge management systems and platforms to ensure effective data utilization and support data-driven decision-making. | ST3 |
|  |  | Address data privacy concerns to increase access to data and information, leveraging the opportunities for revenue streams and new partnerships. | WO2 |
|  |  | Develop policies that address talent shortage through investment in education and training programs, minimizing the weakness of upskilling and avoiding the threat of talent shortage. | WT4 |
|  |  | Develop policies that account for economic uncertainty and global instability in supply chain strategies, minimizing the weakness of infrastructure and avoiding the threat of supply chain balancing. | WT5 |

| Very Poor | Poor | Relatively Good | Good | Very Good | **Verbal Expressions** |
| --- | --- | --- | --- | --- | --- |
| e | d | c | b | a | Points |
